# Effect of Mechanical Damage on Tritium Permeability Resistance of FeAl/Al_2_O_3_ Coating on 316L Stainless Steel

**DOI:** 10.3390/ma17215195

**Published:** 2024-10-25

**Authors:** Yinghong Li, Lihong Nie, Fantao Meng, Haixiang Hu, Sifan Zong, Zhihao Hong, Long Wang, Changzheng Li, Qisen Ren, Jing Hu

**Affiliations:** 1 China Nuclear Power Technology Research Institute Co., Ltd., Shenzhen 518000, China; liyinghong@cgnpc.com.cn (Y.L.); nielihong@cgnpc.com.cn (L.N.); huhaixiang@cgnpc.com.cn (H.H.); lichangzheng@cgnpc.com.cn (C.L.); renqisen@cgnpc.com.cn (Q.R.); 2 College of Materials and Chemistry & Chemical Engineering, Chengdu University of Technology, Chengdu 610059, China; zongsf2024@163.com; 3 Southwestern Institute of Physics, Chengdu 610225, China; mengft2000@163.com (S.Z.); hongzhihao@swip.ac.cn (Z.H.); 4 School of Mechanical Engineering, Chengdu University, Chengdu 610106, China; 5 Polytechnic Institute, Zhejiang University, Hangzhou 310015, China

**Keywords:** FeAl/Al_2_O_3_ tritium permeation barrier, mechanical damage, tritium permeation

## Abstract

Depositing a tritium permeation barrier on the surface of materials is a key method for reducing tritium permeability. During actual operational processes, the surface of the tritium permeation barrier may experience mechanical damage, such as spalling and scratches. The hydrogen permeability resistance of the coating will degrade due to such forms of mechanical damage. It is a significant engineering challenge to evaluate the impact of these damages on the coating’s tritium resistance. In this experiment, the mechanical damage to the FeAl/Al_2_O_3_ tritium permeation barrier on 316L stainless steel was simulated through scratching, debonding, and thermal shock. Subsequently, a hydrogen isotope gas drive permeation (GDP) test was conducted. The influence of the degree of mechanical damage on the coating’s tritium permeation behavior was assessed and discussed. The results indicate that, under the same damage mechanism, the coating’s tritium permeability resistance is positively correlated with the integrity of the coating. Additionally, the impact of scratches on the coating surface is more severe than that of other damage mechanisms.

## 1. Introduction

The treatment and storage of nuclear waste is a prominent problem in current nuclear energy applications [1]. The tritium-containing wastewater generated during reactor operation must be treated before it can be discharged into the environment. As a hydrogen isotope, tritium has a small atomic radius and high chemical activity. Its permeation and retention in the reactor material are almost unavoidable. The tritium in the reactor is mainly derived from three sources [2,3,4]: the fission reaction of ^235^U, the neutron reaction of the antimony–beryllium pellet in the secondary neutron source rod, and the neutron reaction of boron and lithium in the primary coolant. To reduce tritium leakage in reactors, an effective method is to keep tritium trapped in the fuel cladding material or structural components of the reactor, and the most economical and feasible method is to apply a tritium barrier coating to the surface of the material.

There are many types of tritium permeation barriers, among which ceramic coatings are one of the key research and development directions because of their excellent tritium permeation resistance, good corrosion resistance, and mechanical properties. Ceramic coatings include nitride coatings, silicide coatings, and oxide coatings. Nitride coatings (TiN, CrN, etc.) have a permeability reduction factor (PRF) of up to 10^3^ [5,6], but they are susceptible to oxidation at high temperatures. Silicide coatings, such as SiC, have excellent corrosion resistance and a PRF of about 10^2^ [7]. Oxide coatings are the earliest type of tritium permeation barrier that has been studied and have a wide range of applications and research bases. Typical oxide coatings include Cr_2_O_3_ [8], Er_2_O_3_ [9], and Al_2_O_3_ [10,11,12,13,14], and PRFs can reach 10^2^~10^3^. Among them, Al_2_O_3_ behaves well in terms of tritium permeation resistance, and the PRF can reach more than 10^3^ [15,16]. However, it has the problem of easy peeling and difficulty in preparing the α-Al_2_O_3_ phase, which has the best tritium resistance performance [13].

In this study, to prevent the peeling of the Al_2_O_3_ coating, the FeAl/Al_2_O_3_ composite coating was investigated [17,18]. A FeAl transition layer was formed between the Al_2_O_3_ and the metal substrate, which could mitigate the thermal mismatch between the Al_2_O_3_ coating and the substrate, thereby endowing the coating with a certain self-healing capability. FeAl/Al_2_O_3_ coatings exhibit excellent mechanical properties (including hardness, wear resistance, and resistance to thermal shock properties), can prevent material oxidation and sulfidation [19], inhibit material corrosion and carburization [20,21,22], and effectively block tritium permeation [23,24]. These characteristics make the coating a promising candidate for high-temperature applications.

In the actual manufacturing process and application process in the nuclear reactor, such as welding, transportation, and lifting, the tritium permeation barrier may sustain mechanical damage due to collisions or thermal shock. This damage can affect the coating’s resistance to tritium penetration. Consequently, it is essential to investigate the influence of coating damage on its tritium penetration resistance to guide engineering applications. In this paper, based on the FeAl/Al_2_O_3_ coating on the surface of stainless steel, the different degrees of mechanical damage on the coating and thermal shock are simulated, and the changes in tritium permeability under these different conditions are tested and evaluated using a gas-phase hydrogen isotope permeation test platform.

## 2. Experimental Methods

### 2.1. Coating Preparation

**Figure 1:**
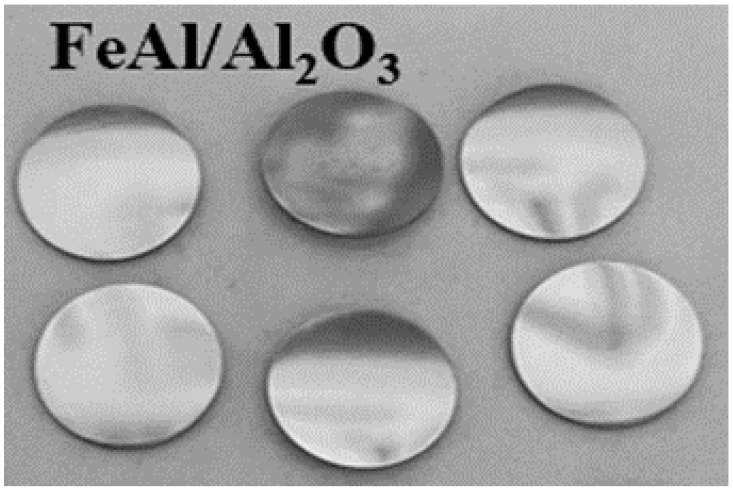
The prepared FeAl/Al_2_O_3_ coating samples.

All 316L stainless steel substrates were processed in the same batch to eliminate the effects of anisotropy [25,26]. The FeAl/Al_2_O_3_ coating was prepared on 316L stainless steel substrate using RF magnetron sputtering equipment (QX-500-2/D, Chengdu, China) The specific operation steps are as follows. The polished sample was placed in the sputtering chamber, which was then vacuumed with the hatch door sealed to create a vacuum. Upon achieving the required vacuum, argon gas was introduced for the brightening process. Once brightening was complete, the parameters were adjusted to the preset values for sputtering. Initially, a FeAl transition layer was deposited on the substrate by adjusting the Fe:Al ratio in the target, with the area ratio of the Fe target to the Al target in the sputtering process being approximately 3:2 [27]. Subsequently, an Al_2_O_3_ ceramic coating was deposited via reactive sputtering in an Ar+O_2_ atmosphere using an Al target. To minimize the impact of impurities on the coating, all targets and gases were of 99.999% purity. The structure of the deposited coating is shown in Figure 1. The coating sample was in the form of a disc with a thickness of 0.5 mm and a diameter of 12 mm. The preparation parameters were based on previous work, with an air pressure of 0.5 Pa, a power of 200 W, and a temperature of 200 °C [27]. The hardness of the coating was 5.35 GPa, and the elastic modulus was 127.22 GPa. The maximum scratch load was 14 N.

### 2.2. Hydrogen Isotope Gas Drive Permeation (GDP) Test

**Figure 2:**
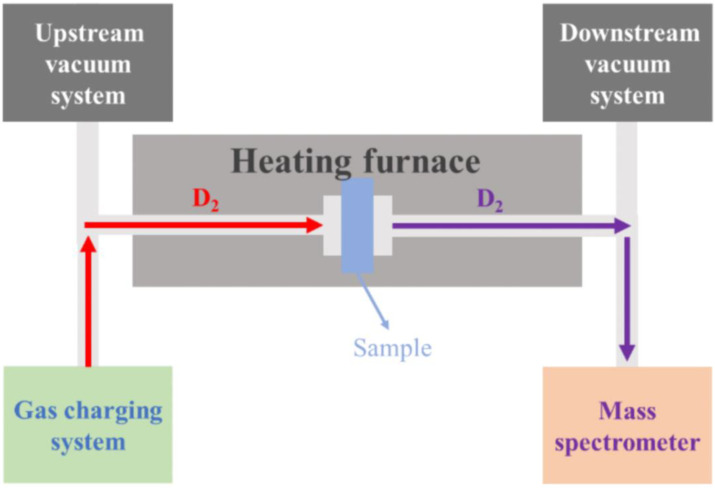
Structure of gas hydrogen isotope permeation test device.

Since tritium is radioactive, its isotope deuterium is used for permeation experiments. The structure of the device is shown in Figure 2. The sample is clamped between the upstream and downstream using a VCR (Vacuum Coupling Radius) seal, with the coated side facing the upstream. Ionization gauges are employed for pressure measurements. The sensitivity of the ionization gauge is 1 × 10^−7^ Pa. To eliminate interference, the device was vacuumed prior to the experiment, achieving a vacuum of 1 × 10^−4^ Pa in the upstream pipeline and 1 × 10^−6^ Pa in the downstream pipeline. During the experiment, deuterium gas was introduced into the upstream pipeline at specific pressures (100 kPa and 500 kPa). The temperature was controlled using a high-temperature furnace, and measurements were conducted at 500 °C, 450 °C, and 400 °C. The deuterium permeation current signal was measured by a mass spectrometer at the downstream detection end.

### 2.3. Microstructure Inspection

After the deuterium permeation test, the microstructure and topography of the sample surface and cross-section were characterized using a Thermo Scientific Apreo 2c model field emission scanning electron microscope (FEEM, Waltham, MA, USA).

### 2.4. Simulation of Mechanical Damage of Coating Surface

Being scratched by sharp objects, peeling due to weak adhesion, and damage from thermal cycling are the common types of mechanical damage in practical applications. To simulate the effects of different damage mechanisms and degrees on the permeability of FeAl/Al_2_O_3_ coatings, three types of damage were simulated:

- Scratch damage: A scratch meter was used to create the linear groove-like damage on the surface;
- Debonding (or spalling) damage: A spatula was employed to create the regional loss of coating on the surface;
- Thermal shock damage: The samples were subjected to 30 thermal shock cycles, with temperatures ranging from room temperature to 450 °C. Both the heating and cooling rates exceeded 500 °C/s.

**Figure 3:**
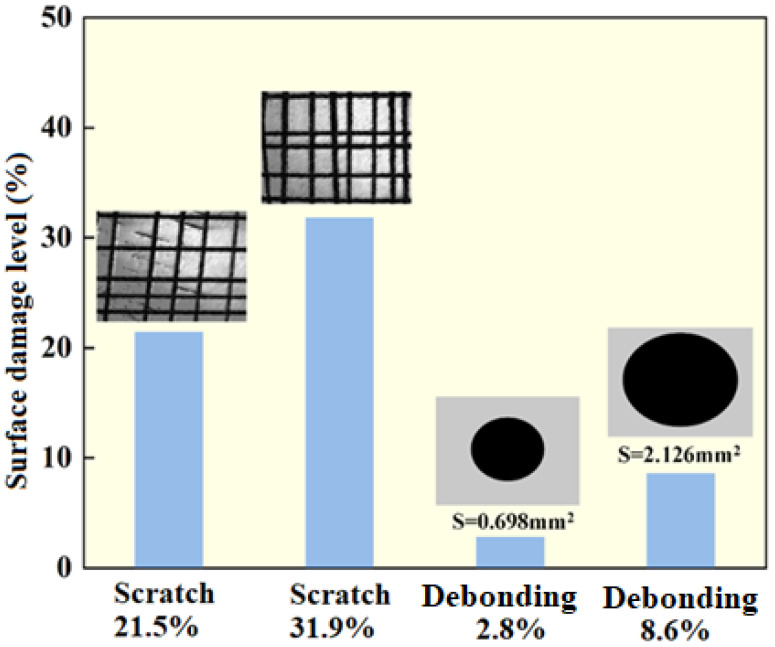
Scratch and debonding damage simulation and surface damage ratios.

The simulated damage ratios are shown in Figure 3.

## 3. Results and Analysis

### 3.1. Hydrogen Isotope Permeation

**Figure 4:**
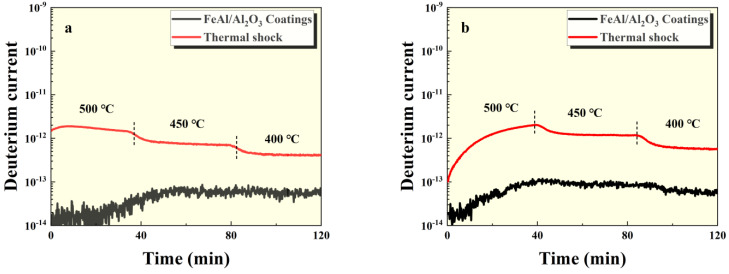
GDP results of FeAl/Al_2_O_3_ coating after thermal shock at (**a**) 100 kPa and (**b**) 500 kPa pressures.

**Figure 5:**
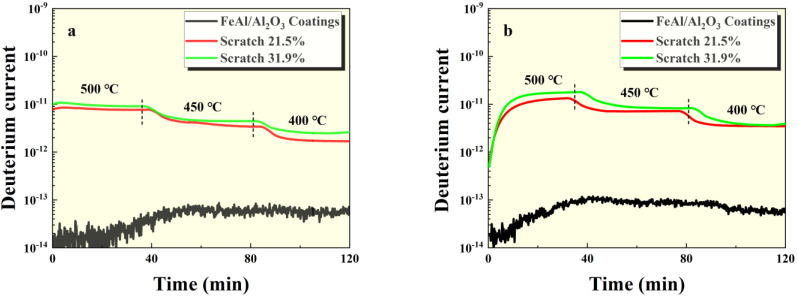
GDP results of FeAl/Al_2_O_3_ coating with scratches at (**a**) 100 kPa and (**b**) 500 kPa pressures.

**Figure 6:**
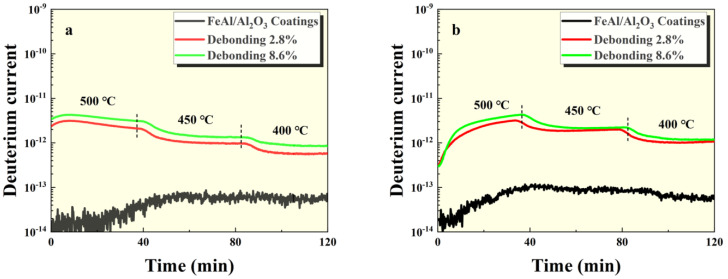
GDP results of FeAl/Al_2_O_3_ coating with debonding at (**a**) 100 kPa and (**b**) 500 kPa pressures.

The GDP test results of the intact FeAl/Al_2_O_3_ coating sample and the damaged sample at 100 kPa and 500 kPa pressures are shown in Figure 4, Figure 5 and Figure 6. It can be seen that the permeation signal of the intact sample was maintained at the order of 1 × 10^−13^, signifying its excellent resistance to tritium permeation. In comparison, the permeation signal of the thermal shock, scratch, and debonding damage samples increased by two, three, and two orders of magnitude, respectively, compared to the intact sample. This indicates that any form of mechanical damage significantly impairs the coating’s tritium permeation resistance. Notably, scratches have a particularly severe impact on the coating’s performance. Furthermore, as depicted in Figure 5 and Figure 6, the greater the degree of surface damage to the coating, the more compromised its tritium resistance becomes, which is consistent with expectations.

**Figure 7:**
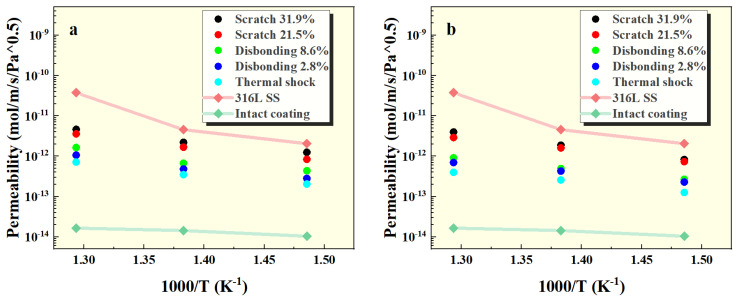
Permeability of FeAl/Al_2_O_3_ coatings at (**a**) 100 kPa and (**b**) 500 kPa pressures.

Based on the permeation signal measurements, the permeability of the samples at different temperatures was calculated (as shown in Table 1 and Table 2) and compared with that of 316L stainless steel, both with and without the intact coating, as shown in Figure 7. The results indicate that the intact coating can reduce the tritium permeability by two to three orders of magnitude compared to the uncoated 316L stainless steel. Coatings with missing integrity still exhibit some resistance to tritium penetration. Additionally, the data reveal that the permeability at 100 kPa is slightly higher than that at 500 kPa, which may be due to the presence of the coating, making the surface reaction process of tritium atoms lower than bulk diffusion.

### 3.2. Microstructure Examination Results

#### 3.2.1. Intact Coating

**Figure 8:**
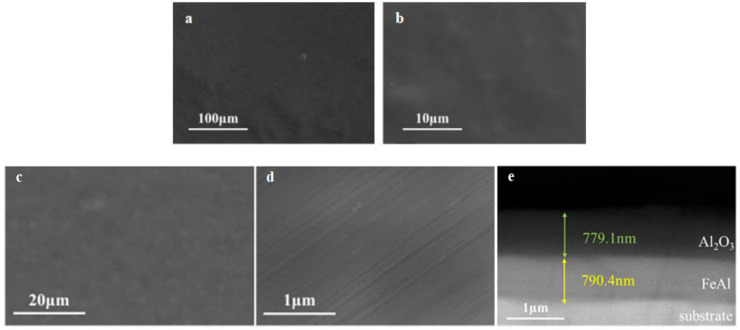
SEM results of the intact FeAl/Al_2_O_3_ coating, (**a**,**b**) before GDP test, (**c**–**e**) after GDP test.

The microstructure examination results of the intact FeAl/Al_2_O_3_ coating are shown in Figure 8. It shows that the prepared FeAl/Al_2_O_3_ coating exhibits high surface flatness and integrity. After the deuterium gas penetration test at high temperature, the surface of the coating remained flat, with no evident peeling or cracking observed. The cross-sectional image reveals that the coating is layered, and the interface remains intact, indicating that the coating has good high-temperature stability.

#### 3.2.2. Coating after Thermal Shock

**Figure 9:**
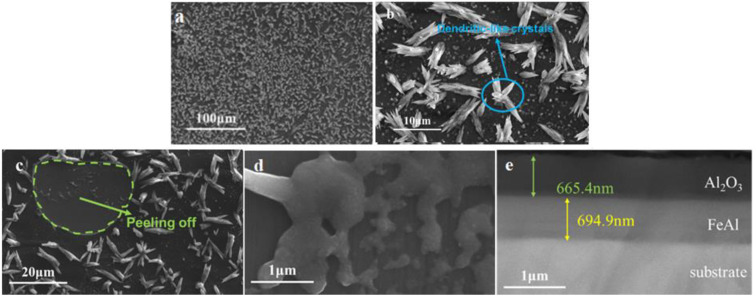
SEM results of FeAl/Al_2_O_3_ coating after thermal shock, (**a**,**b**) before GDP test, (**c**–**e**) after GDP test.

The microstructure examination results of the FeAl/Al_2_O_3_ coating after thermal shock are shown in Figure 9. It shows that there is no obvious cracking on the surface of the coating after thermal shock, but some dendritic-like crystals were produced on the surface. These crystals persisted after deuterium penetration, but the coating surface exhibited peeling. The spalled area is flat, revealing a white lamellar structure inside, which is formed by cluster-like aggregation and could be produced after the coating is peeled off. The cross-sectional results showed that the coating structure remained intact after thermal shock, with no visible damage to the interior. Based on the above results, it is judged that the spalling of the outer Al_2_O_3_ layer after thermal shock is attributed to the difference in the thermal expansion coefficient between the coating and the substrate. And as the spalling is not affected by mechanical forces, the affected area of the coating remains relatively flat.

The microstructure examination results indicate that the enhanced tritium permeability after thermal shock is primarily due to the spalling of the Al_2_O_3_ layer on the surface. Moreover, the formation of dendritic-like crystals on the surface also increases the area available for deuterium gas adsorption, thereby facilitating the tritium penetration process.

#### 3.2.3. Coating with Scratch

**Figure 10:**
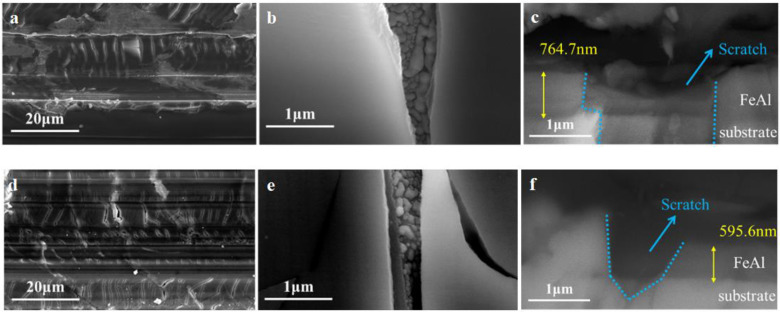
SEM results of FeAl/Al_2_O_3_ coating with (**a**–**c**) scratch 21.5% and (**d**–**f**) scratch 31.9% after the GDP test.

SEM results of the FeAl/Al_2_O_3_ coating with a scratch after the GDP test are shown in Figure 10. It is evident that the scratch area is riddled with numerous cracks. The internal morphology of these cracks is irregular, exhibiting a roughness greater than that of the coating surface. This structure will be more conducive to the adsorption and dissociation of tritium molecules, thus enhancing permeation. Furthermore, the cross-sectional results reveal that the scratches have significantly damaged the coating. Both the outer Al_2_O_3_ layer and the underlying FeAl layer have sustained damage. The scratch depth extends to the substrate surface, exposing the substrate and enabling direct tritium penetration through this area. Consequently, the coating’s resistance to tritium permeation has been diminished.

#### 3.2.4. Coating with Debonding

**Figure 11:**
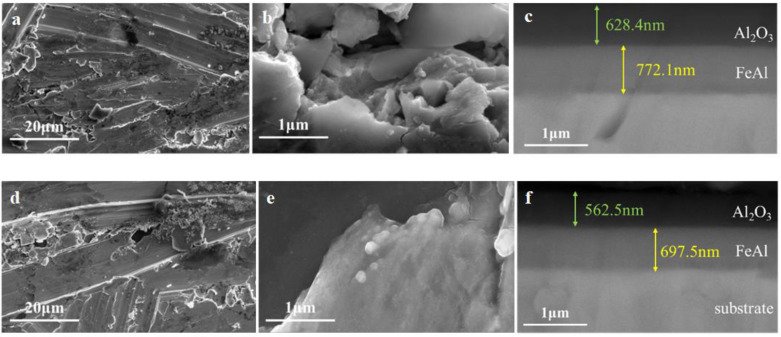
SEM results of FeAl/Al_2_O_3_ coating with (**a**–**c**) 2.8% debonding and (**d**–**f**) 8.6% debonding after the GDP test.

Figure 11 depicts the SEM results of the FeAl/Al_2_O_3_ coating with debonding after the GDP test. The topography of the coating surface at the site of debonding appears uneven, with the presence of some small potholes and fractured edges around the holes. The cross-sectional inspection results indicate that the interface structure of the coating remains intact, with no exposure to the substrate. In conjunction with the results of the permeation experiment, this suggests that the retained FeAl layer continues to function effectively in preventing tritium penetration.

## 4. Summary

In this paper, the tritium resistance performance of FeAl/Al_2_O_3_ coatings with different mechanical damage was evaluated by carrying out coating damage simulation and hydrogen isotope gas drive permeation (GDP) test. And the microscopic examination was conducted to analyze the potential causes of changes in tritium resistance performance following coating damage.

The test results confirm a correlation between the tritium permeation resistance of the FeAl/Al_2_O_3_ coating and its structural integrity. The intact FeAl/Al_2_O_3_ coating exhibits excellent tritium resistance, approximately three orders of magnitude. However, once the coating’s integrity is compromised, its tritium resistance performance diminishes significantly. The greater the degree of coating damage, the poorer the tritium resistance performance. In the most severe case, the tritium penetration resistance of the coating is almost completely lost.

Microstructure analysis reveals that the tritium resistance of FeAl/Al_2_O_3_ coating is influenced by both the surface and cross-sectional conditions of the coating. An uneven coating surface or the presence of dendritic-like crystals or rough cracks within the coating increases the surface area available for tritium adsorption and dissociation, facilitating the tritium penetration process. If the outer Al_2_O_3_ layer of the coating peels off, the barrier effect against tritium penetration in the affected area is greatly reduced. Furthermore, if the internal FeAl layer is also damaged, the substrate becomes directly exposed, allowing tritium to penetrate through this area and rendering the coating ineffective as a tritium barrier.

Therefore, to maintain the tritium resistance performance of FeAl/Al_2_O_3_ coating, it is crucial to preserve the coating’s integrity as much as possible during the actual manufacturing and application process.

## Figures and Tables

**Table 1:** Permeability of FeAl/Al_2_O_3_ coatings at 500 kPa.

Temperature/°C	Permeability (mol/m/s/Pa^0.5^)				
	Scratch 31.9%	Scratch 21.5%	Debonding 2.8%	Debonding 8.6%	Thermal Shock
500	4.0 × 10^−12^	2.9 × 10^−12^	9.1 × 10^−13^	6.9 × 10^−13^	4.0 × 10^−13^
450	1.9 × 10^−12^	1.6 × 10^−12^	4.9 × 10^−13^	4.3 × 10^−13^	2.6 × 10^−13^
400	8.3 × 10^−13^	7.4 × 10^−13^	2.7 × 10^−13^	2.3 × 10^−13^	1.3 × 10^−13^

**Table 2:** Permeability of FeAl/Al_2_O_3_ coatings at 100 kPa.

Temperature/°C	Permeability (mol/m/s/Pa^0.5^)				
	Scratch 31.9%	Scratch 21.5%	Debonding 2.8%	Debonding 8.6%	Thermal Shock
500	4.6 × 10^−12^	3.6 × 10^−12^	1.6 × 10^−12^	1.1 × 10^−12^	7.2 × 10^−13^
450	2.2 × 10^−12^	1.7 × 10^−12^	6.7 × 10^−13^	4.8 × 10^−13^	3.5 × 10^−13^
400	1.3 × 10^−12^	8.4 × 10^−13^	44 × 10^−13^	2.8 × 10^−13^	2.0 × 10^−13^

## Data Availability

The original contributions presented in the study are included in the article, further inquiries can be directed to the corresponding authors.
